# The effect of various concentrations of myo-inositol in culture medium on development of bovine embryos

**Published:** 2012-09

**Authors:** Leila Soltani, Farokh Kafilzadeh, Hamed Karami Shabankareh

**Affiliations:** *Department of Animal Sciences, Faculty of Agriculture, Razi University, Kermanshah, Iran.*

**Keywords:** *Bovine*, *Myo-inositol*, *Fertilization*, *Embryo development*

## Abstract

**Background:** Progress achieved in culture media formulations have resulted led to an improvement in maintaining the mammalian embryo in culture throughout the preimplantation and pre-attachment period.

**Objective:** The objective of this study was to evaluate the effect of various concentrations of myo-inositol during in vitro fertilization of bovine oocytes on subsequent embryo development.

**Materials and Methods:** Bovine cumulus oocytes complexes (COCs) were matured in vitro at 39^o^C, in humidified 5% CO_2_ atmosphere for 22-24 h. The COCs were co-incubated with epididymal spermatozoa of post mortem bulls in modified TALP medium for 22-24hr. The fertilization medium used was: 1) TALP medium without myo-inositol (control); 2) control+0.02 g/l myo-inositol; 3) control+0.03 g/l myo-inositol; 4) control+0.04 g/l myo-inositol. Zygotes were cultured in vitro for 8 days when the ratios of in vitro embryo development of the hatched blastocysts were assessed and compared with the control group (p<0.05).

**Results:** The presence of 0.04 g/l myo-inpsitol significantly improved overall morula and blastocyst rates (46.94%) compared to control (32.19%), but there was no difference in the percentage of embryos successfully developed to the morula and blastocyst stage when different levels of myo-inositol were used (46.94, 36.36 and 37.33% respectively). The mean percentage of cleavage rate was not significantly affected by treatments.

**Conclusion:** These results suggest that, addition of 0.04 g/l myo-inositol in TALP medium is more beneficial for subsequent bovine embryonic development.

## Introduction

Myo-inositol is formed from glucose-1-phosphate in a NAD-catalyzed oxidation/ reduction reaction. It is a cyclic alcohol with 6 hydroxyl groups, one of many stereoisomers (1). Myo-inositol plays an important role in cell morphogenesis and cytogenesis, lipid synthesis, structure of cell membranes and cell growth (2). 

The effects of vitamins have been tested on oocytes maturation and preimplantation embryo culture systems and some benefits on oocytes maturation and embryo development have been observed. The addition of inositol, choline and pantothenate to chemically defined medium, improved hatched blastocyst of hamsters embryos (3). 

Rabbit blastocysts required inositol, pyrodoxal, riboflavin and niacinamide for expansion and growth. The most drastic reduction in expansion was caused by the omission of inositol (4). Inositol is essential for rabbit blastocyst expansion, cell proliferation and protein synthesis and show that the effect on rabbit blastocyst expansion (5). 

Addition of citrate and myo-inositol improved blastocyst development rates in defined medium to comparable to those obtained with serum, BSA or co-culture (6). Improvement in preimplantation embryo development has been reported with myoinositol uptake from culture medium in studies with a number mammalian species (7, 8). 

However, on the basis of our knowledge, there is not much information about the effect of various concentrations of myo-inositol on in vitro fertilization and embryo development in bovine. The objective of this study was to determine if supplementation of various concentrations of myo-inositol in TALP fertilization medium could subsequent in vitro embryo development.

## Materials and methods

**Chemicals**

Unless mentioned otherwise, growth factors and other chemicals were purchased from Sigma (USA), plastics from Falcon (UK), hormones (eCG and hCG) from Intervet (Netherlands). All the media were incubated at 39^o^C under humidified atmosphere of 5% CO_2_ in air for 1 hr prior to use.

**Oocyte collection**

Ovaries were transported from the local abattoir to laboratory in 0.9% saline at 30-35^o^C. They were washed three times with warmed 0.9% saline. COCs were aspirated from 2-6 mm follicles using a 20-gauge needle attached to a 10 ml syringe. COCs were collected in TCM HEPES medium supplemented with 14.24 mg/ml heparin and 50 g/ml gentamicin.

**In vitro maturation**

COCs with at least three layers of nonexpanded cumulus and homogeneous cytoplasm were selected and washed three times in TCM HEPES and once in TCM-199 maturation medium supplemented with 0.23 mmol/l sodium pyruvate, 50 ng/ml epidermal growth factor (EGF), 10 IU/ml equine chorionic gonadotrophin (eCG), 10 IU/ml human chorionic gonadotrophin (HCG) and penicillin/streptomycin (100 U/ml penicillin, 100 g/ml streptomycin). Oocytes were matured in groups of 10 per 50 l droplets in culture dishes under 10 ml mineral oil. Maturation proceeded for 22-24h at 39^o^C and 5% CO_2_ in air with 100% humidity (9). 

**In vitro fertilization**

At the end of the maturation period, oocytes were briefly placed in TCM199 maturation medium supplemented with 500 IU/ml hyaluronidase, followed by gentle pipeting to dissociate surrounding cumulus cells and washed three times in fertilization medium. 

Oocytes (10-15) were placed into a 100 µl drop to which sperm cells were added 5 min later. An epididymal spermatozoa was collected from post mortem bulls and kept at room temperature for up to 2 h, then washed in TALP, centrifuged twice at 200×g for 5 min. The medium used for in vitro fertilization was TALP, supplemented with 6 mg/ml of BSA-FFA, 0.23 mmol/l sodium pyruvate, 10 µg/l heparin, 20 µmol/l penicillamine, 10 µmol/l hypotaurine, 1 µmol/l epinephrine (PHE) as originally described by Parrish *et al* (10), in this study sperms and oocytes co-incubated without or with different concentration of myo-inositol. 

Treatment groups were: I) TALP medium without myo-inositol (Control); II) Control+0.02 g/l myo-inositol; III) Control+0.03 g/l myo-inositol; IV) Control+0.04 g/l myo-inositol. Sperms were counted in a hemacytometer and checked for acceptable motility (i.e., at least 80% progressively motile); then oocytes were inseminated by addition of sperm to result in a final concentration of 1.0×10^6^ motile sperm per milliliter. Sperm and oocytes were incubated under mineral oil at 39^o^C under humidified atmosphere of 5% CO_2_ for 22-24h. 

**In vitro culture**

Embryo culture took place in modified SOF under mineral oil in a humidified atmosphere of 5% CO_2_ and 5% O_2_ and 90% N_2_ at 39^o^C. Between 15 and 22-24 h after insemination, presumptive zygotes were denuded of surrounding cumulus cells by repeated pipetting in SOF HEPES and subsequently washed three times in SOF HEPES before being transferred to the culture droplets (50 µl) in groups of 10-15 embryos. Cleavage was assessed after 48 h of culture, and the number of embryos developing to the morula and blastocyst stages was assessed on day 8.

To prevent toxic accumulation of ammonium as a result of amino acid degradation, SOF medium was replaced every 48 h. In this study, we used a two-culture system. The first system (SOFC1) medium contained 0.8% BSA crystallized, MEM nonessential amino acids, 1 mM glutamine, 1.5mM glucose, and 10 µM EDTA for the first 48 h. Then, the medium was replaced by the second system (SOFC2) containing 0.8% BSA crystallized, MEM nonessential amino acids, MEM essential amino acids, 3mM glucose, and 1mM glutamine for the remaining 6 days of culture (9). 

**Statistical Analysis**

This experiment was replicated 5 times and COCs were randomly allocated into each treatment group. The data were analyzed by one-way ANOVA using SPSS (11.5, 2004) program. Duncan s multiple range test was used to test the differences between treatments. P-value <0.05 indicated as a significant difference.

## Results

In the present study, there were no differences between treatments in the percentage of embryos successfully developing to the cleavage stage, expressed as a percentage of total inseminated oocytes (Table I). The presence of 0.04 g/l myo-inositol significantly increased the percentage of morula and blastocyst (p<0.05) as compared with control (46.94 and 32.19; respectively, Table I). No significant difference was also observed in morula and blastocyst rates between 0.02, 0.03 and 0.04 g/l myo-inositol (36.36%, 37.33% and 46.94%; respectively).

**Table I:** Effect of different concentrations of myo-inositol added during IVF of bovine oocytes on subsequent embryonic development.

**Treatments** *	**Oocytes (n)**	**Percentage cleaved**	**Morula and blastocyst/ cleaved (%)** **
Treatments I	135	66.55 ± 4.35	32.19±3.49^b^
Treatments II	120	66.66 ± 4.42	36.36±2.35^ab^
Treatments III	128	61.38 ± 4.45	37.33±2.98^ab^
Treatments IV	117	62.59 ± 4.83	46.94±6.48^a^

Values within a column with different superscripts (a, b) are significantly different, (p<0.05).

Treatments II: Control + 0.02 g/l myo-inositol.

Treatments III: Control + 0.03 g/l myo-inositol.

Treatments IV: Control + 0.04 g/l myo-inositol.

* Treatments I: TALP medium without myo-inositol (Control).

** Sum of the morula and blastocysts per cleaved oocytes.

## Discussion

Myo-inositol has long been known as an essential growth factor for mammalian cells (11). Inositol is essential for the synthesis of certain membrane phospholipids in cells and it is possible that at the blastocyst stage it becomes necessary for the synthesis of phospholipid components of new membranes (12, 13). Inositol may play an essential role in the formation of pronuclei during in vitro fertilization (3). Rabbit blastocysts required inositol, pyridoxal, riboflavin and niacinamide for expansion and growth (4, 14). 

Kane showed that the omission of inositol, pyridoxine, riboflavin, and niacinamide resulted in large statistically significant decreases in blastocyst expansion but omission of B_12_ resulted in a significant increase in blastocyst expansion indicating that the level present in F10 is toxic to rabbit blastocysts (14). Funahashi *et al* reported that the addition of myo-inositol in maturation medium of procine oocytes had no significant effect on cytoplasmic maturation of oocytes (15). 

They concluded that, this may be due to a difference in the effective order because supplementation with 12 mM myo-inositol increased the osmolality of maturation medium by only 6 mOsM (milli osmole). In the present study, the addition of myo-inositol (0.04 g/l) in fertilization medium (TALP) containing BSA significantly increased blastocyst development as compared with control. 

This finding did not agrees with previous data from Holm *et al* who reported no effect of myo-inositol on blastocyst development in SOF containing citrate and BSA (6). Nevertheless, these author a stimulating effect of myo-inositol in medium containing polyvinyl alcohol (PVA) and reported that minute concentrations of myo-inositol could be present in commercial BSA preparations. Another study reported there was higher developmental ability of embryos cultured in chemically defined medium with myo-inositol, EGF and modification of energy substrate composition (16). 

Kane used high concentrations (75 m) of myo-inositol to culture medium and found a significant effect on rabbit blastocyst expansion (17). Fahy *et al* indicated that inositol is essential for rabbit blastocyst expansion, cell proliferation and protein synthesis (18). Chiu *et al* showed that, myo-inositol has a positive effect on meiotic maturation and developmental competence of maturing mouse oocytes in the absence of exogenous gonadotrophins (19). In addition, previous work a from our laboratory showed that addition of myo-inositol as compared to MEM vitamins may have beneficial effect on blastocyst rate in sheep (8). 

Results from the present study agree with previous works showing that the addition of myo-inositol to maturation and culture medium increased blastocyst rate. In conclusion, present study indicated that under the defined conditions, responses to supplementation of various concentrations of myo-inositol to fertilization medium depended on the stage of embryo development (3, 4, 14, 19). 

The presence of 0.04 g/l myo-inositol in fertilization medium increased morula and blastocyst rate as compared to control groups. It is possible, lower concentrations of myo-inositol were ineffective or not sufficient to IVF and subsequent embryonic development. However, further studies needed to determine whether the presence of myo-inositol in fertilization medium in high concentrations could further enhance fertilization and subsequent embryo development or not.
